# Epidemiology of Major Depressive Disorder in Mainland China: A Systematic Review

**DOI:** 10.1371/journal.pone.0065356

**Published:** 2013-06-13

**Authors:** Lian Gu, Juanjuan Xie, Jianxiong Long, Qing Chen, Qiang Chen, Runde Pan, Yan Yan, Guangliang Wu, Baoyun Liang, Jinjing Tan, Xinfeng Xie, Bo Wei, Li Su

**Affiliations:** 1 Department of Internal Neurology, First Affiliated Hospital, Guangxi University of Chinese Medicine, Nanning, Guangxi, China; 2 School of Public Health of Guangxi Medical University, Nann-ing, Guangxi, China; 3 Guangxi Brain Hospital, Liuzhou, Guangxi, China; The University of Hong Kong, Hong Kong

## Abstract

**Background:**

Major depressive disorder (MDD) is one of the important causes of disease burden in the general population. Given the experiencing rapid economic and social changes since the early 1990s and the internationally recognized diagnostic criteria and interview instruments across the surveys during 2001–2010 in china, the epidemiological studies on MDD got varied results. We performed this meta-analysis to investigate current, 12-month and lifetime prevalence rates of MDD in mainland China.

**Methods:**

PubMed, Embase, Chinese Biological Medical Literature database (CBM), Chinese National Knowledge Infrastructure database (CNKI), and the Chinese Wanfang and Chongqing VIP database were searched for associated studies. We estimated the overall prevalence of MDD using meta-analysis.

**Conclusions:**

Seventeen eligible studies were included. Our study showed that the overall estimation of current, 12-month and lifetime prevalence of MDD was 1.6, 2.3, 3.3%, respectively. The current prevalence was 2.0 and 1.7% in rural and urban areas, respectively; between female and male, it was 2.1 and 1.3%, respectively. In addition, the current prevalence of MDD diagnosed with SCID (Structured Clinical Interview for DSM-IV) was 1.8% and that diagnosed with CIDI (Composite International Diagnostic Interview) was 1.1%. In conclusion, our study revealed a relatively high prevalence rate in the lifetime prevalence of MDD. For current prevalence, MDD diagnosed with SCID had a higher prevalence rate than with CIDI; males showed a lower rate than females, rural residents seemed to have a greater risk of MDD than urban residents.

## Introduction

Major depressive disorder (MDD) is considered to be the most common mental illness, and it is the fourth primary cause of disability with annual total costs to society exceeding $80 billion worldwide [1]–[6]. Moreover, it is predicted that MDD will be the second-highest cause of global morbidity and mortality by 2020 [7]. Some studies reported that in North America and Western Europe, the prevalence rate of MDD in the previous year or in the previous month fluctuated within a range of 2 and 6% in the adult general population [8]–[13]. However, the lifetime prevalence of MDD was 3.5% [14] and the 12-month MDD prevalence was 2% in China [15].

China is one of the middle-income countries that has been experiencing rapid economic and social changes since the early 1990s, leading to a huge number of social phenomenon such as mounting rates of divorce, alcohol and illicit drug abuse, fast growing costs of health care, weakening of family ties, increased number of farmers migrating to urban areas for temporary jobs and so on [16]. Above all of these with a major potential impact on mental health in general is depression [17]. So far, only two national surveys of mental disorders were conducted in 1982 and 1993 in China [18], [19]. However, both surveys only refer to manic-depressive psychosis [20] and exclude available data related to MDD. Beginning in 2000, the psychiatric epidemiological surveys of mainland China have adopted internationally recognized diagnostic criteria and standardized interview instruments [21]. Consequently, up to now only limited data on Chinese epidemiological studies of MDD can be obtained.

There are also inconsistent results of previous studies about the prevalence of MDD during 2001–2010 in mainland China. The high rates were reported in Zhejiang [22] and Qingdao (Shandong) [23], whereas other lower frequencies of MDD were described in Guangxi [24] and Yibin (Sichuan) [25]. Furthermore, it is vital for the policy-makers and mental health professionals to anticipate the needs of patients with MDD. Thus, the aim of this study was to estimate the prevalence of MDD based on all published available studies from 2001 to 2012, which is the first meta-analysis on the prevalence of MDD in mainland China. The specific objectives were: (1) to determine the prevalence of MDD; (2) to identify the gender, diagnostic tools and area distribution of the patients. This review on the prevalence of MDD will not only improve the mental health care of people at need, but also guide us in preventing psychiatric disorders.

## Materials and Methods

### Literature Search

PubMed, Embase, Chinese Biological Medical Literature database (CBM), Chinese National Knowledge Infrastructure database (CNKI), Chinese Wanfang and Chongqing VIP database were searched using the search term “mental disorder”, “major depression”, “major depressive disorder”, “major depressive episode”, “epidemiology”, “prevalence” and “survey” from Jan. 2000 to July 2012, limited to English and Chinese. We also used reference lists and reviews to find additional studies that are considered relevant to the topic.

### Inclusion and Exclusion Criteria

All identified titles and abstracts were reviewed by two independent reviewers. All of the included studies should meet the following selection criteria: (i) cross-sectional study; (ii) based on general population samples rather than volunteers (iii) participants aged 15 years and over; (iv) cross-sectional study that provided the prevalence of MDD; (v) used a screening instrument in the first phase of the study, such as the Composite International Diagnostic Interview (CIDI), General Health Questionnaire (GHQ). In the second phase of the study, the standardized clinical diagnostic of MDD must be based on internationally recognized standards, such as the Diagnostic and Statistical Manual of Mental Disorders (DSM) series and the International Statistical Classification of Diseases, 10th Revision (ICD-10).

The studies with the following characteristics were excluded: (1) reviews, editorials, letters, commentaries and reports; (2) repeated study in which the data has been already included in another study.

### Data Extraction and Statistical Analysis

Two investigators extracted data independently and any disagreements were resolved by consensus. The information we extracted from the included studies was as follows: name of the first author, the date of investigation, publication year, location, and sample size, age of participants, screening instrument, diagnosis criteria, and prevalence of MDD.

The pooled prevalence of MDD and subgroups were calculated with STATA 11.1 and Review Manager (RevMan) 5.1. The fixed-effects or random-effects model was chosen to calculate the OR and 95%CI rely on I^2^. If I^2^ was beyond 50%, we chose the random model. Subgroup analyses by gender, geographical location, and diagnostic tools were also performed.

## Results

### Search Results

**Figure 1:**
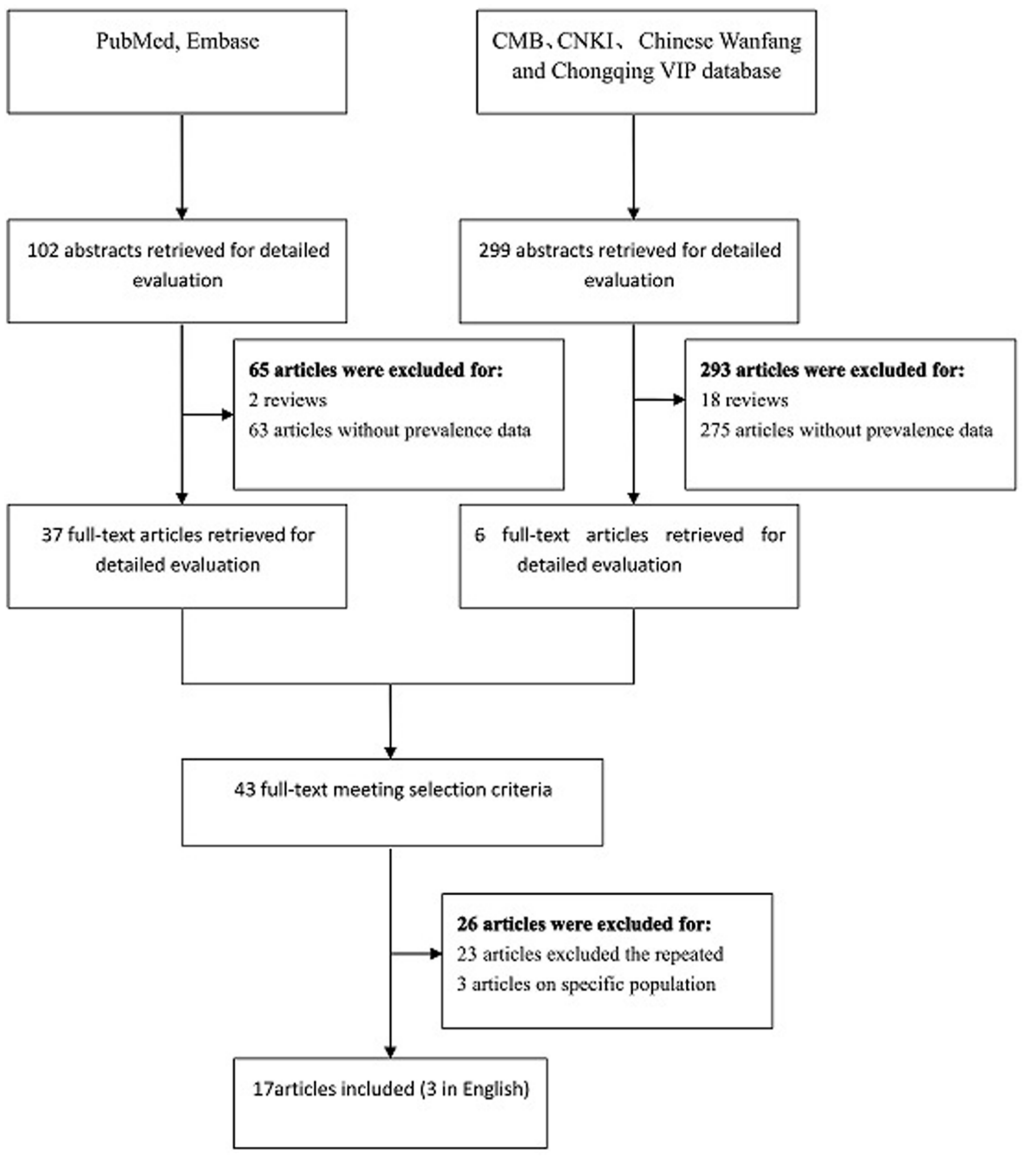
Flow diagram of study identification.

According to the criteria outlined in the Methods section, 401 related articles were identified from all of the databases (Fig. 1). By reading the abstract evaluation of these studies, we excluded the reviews (n = 20), the mental disorder studies that did not provide individual MDD data (n = 338). The repeated studies (n = 23) and the specific population studies (n = 3) based on the full-text were excluded. Finally, 17 studies were included in our study [22]–[38]. No additional articles can be obtained from the reference lists.

### Characteristics of Included Studies

A total of 176,435 subjects from 17 studies were included in the meta-analysis. All included studies were conducted from 2001 to 2010 and covered 12 provinces and two municipalities in mainland China including Beijing(3), Sichuan(1), Fujian(1), Guangxi(1), Hunan(1), Guangdong(2), Yunnan(1), Gansu(1), Qinghai(1), Shandong(2), Liaoning(1), Hebei(1), Zhejiang(1) and Shanghai(1).

Almost all studies have been regulated in two phases, which used GHQ-12 or CIDI as a screening tool in the first phase, and then they were diagnosed by DSM-III or DSM-IV criteria. However, one study was based on ICD10 criteria [24]. Among the included studies, the target population in two studies lived in urban areas [30], [37], and in one study lived in a rural area [38], while the remaining studies had recruited samples from both urban and rural areas. In addition,eight studies have reported the lifetime MDD prevalence of 0.5 to 6.0% in different provinces during 2001–2010[24], [28]–[30], [34]–[37], and six studies reported the 12-month prevalence [26], [29], [30], [34], [36], [37]. Fourteen studies reported the current MDD prevalence with a maximum rate of 3.1% in Zhejiang province and a minimum rate of 0.3% in Guangxi province [22]–[25], [27]–[35], [38]. In addition, between these 14 studies, nine studies used the SCIDI diagnosis tool [22], [25], [27], [30]–[33], [35], [38],five studies used the CIDI tool [24], [28]–[30], [34], seven studies provided the individual data from males and females [22], [27]–[30], [35], [38], and four studies contained the available data from rural and urban areas [22], [27], [28], [35]. The characteristics of all studies in the review are shown in Table 1.

**Table 1:** Characteristics of the studies.

First author	Surveydate	U &R*	City(Province)	Age(year)	Instrument/Questionnaire	Sample size(Male/Female)	Case (n)/Prevalence (95%CI) (%)		
							Current	12month	Lifetime
Chen et al. [28]	2010	U &R	Beijing	≥16	CIDI3.0&CIDI 3.0	2,469(967/1,502)	–	81/3.3(2.6–4.0)	–
Zhang et al. [27]	2009–2010	U &R	Yibin(Sichuan)	≥18	SCID &CHQ12	11,227(5,258/5,969)	39/0.3(0.2–0.5)	–	–
Fang et al. [29]	2009	U &R	Fujian	≥15	SCID &CHQ12	9,986(5,079/4,907)	186/1.9(1.6–2.1)	–	–
Wei et al. [26]	2007	U &R	Guangxi	≥15	CIDI3.0&CIDI3.0	18,21(9,196/9,023)	62/0.3(0.3–0.4)	–	97/0.5(0.4–0.6)
Gui et al. [40]	2007	R	Liuyang(Hunan)	≥15	SCID &SCID	7,347(3,358/3,989)	140/1.9(1.6–2.2)	–	–
Zhao et al. [30]	2006	U &R	Guangzhou	≥15	CIDI3.0&SCID	7,418(3,226/4,192)	62/0.8(0.6–1.0)	–	342/4.6(4.1–5.1)
Lu et al. [31]	2005–2006	U &R	Kunming (Yunnan)	≥15	CIDI2.1&CIDI	5,033(2,416/2,617)	42/0.8(0.6–1.1)	57/1.1(0.8–1.4)	79/1.6(1.2–1.9)
Duan et al. [32]	2005	U	Shenzhen	≥18	CIDI3.1 &CIDI	7,134(3,615/3,519)	90/1.3(1.0–1.5)	215/2.4(2.1–2.7)	429/6.0(5.5–6.6)
Pang et al. [25]	2005	U &R	Qingdao(Shandong)	≥18	SCID &CHQ12	4,776(2,211/2,565)	137/2.9(2.4–3.3)	–	–
Ding et al. [33]	2005	U &R	Tianshui(Gansu )	≥18	SCID &CHQ12	10,249(5,519/4,730)	232/2.3(2.1–2.6)	–	–
Song et al. [34]	2005	U &R	Qinghai	≥18	SCID &CHQ12	11,178(5,652/5,526)	100/0.9(0.7–1.1)	–	–
Zhang et al. [35]	2004–2005	U &R	Shandong	≥18	SCID &CHQ12	22,718(10,457/12,261)	289/1.3(1.1–1.4)	–	–
Zhang et al. [36]	2004–2005	U &R	Liaoning	≥18	CIDI 1.0&CIDI 1.0	13,358(6,610/6,748)	313/2.3(2.1–2.6)	245/1.8(1.6–2.1)	345/2.6(2.3–2.9)
Li et al. [37]	2004–2005	U &R	Hebei	≥18	SCID &CHQ12	20,716(10,343/l0,373)	399/1.9(1.7–2.1)	–	608/2.9(2.7–3.2)
Ma et al. [38]	2003	U &R	Beijing	15–64	CIDI 1.0&CIDI 1.0	4,767(2,190/2,577)	–	152/3.2(2.7–3.7)	253/5.3(4.7–5.9)
Lee et al. [39]	2001–2002	U	Beijing Shanghai	18–80	CIDI&CIDI	5,201(2,533/2,668)	–	89/1.7(1.4–2.1)	181/3.5(3.0–4.0)
Shi et al. [24]	2001	U &R	Zhejiang	≥15	SCID &GHQ12	14,639(7,176/7,463)	452/3.1(2.8–3.4)	–	–

U: urban; R: rural.

### Prevalence of MDD

**Figure 2:**
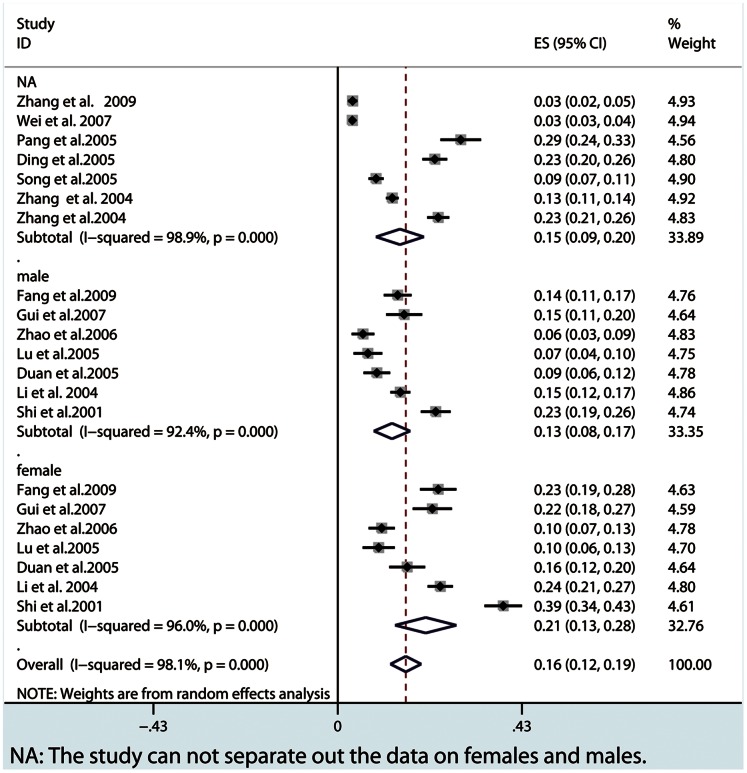
The overall current prevalence in all the MDD patients and subgroups by gender.

**Figure 3:**
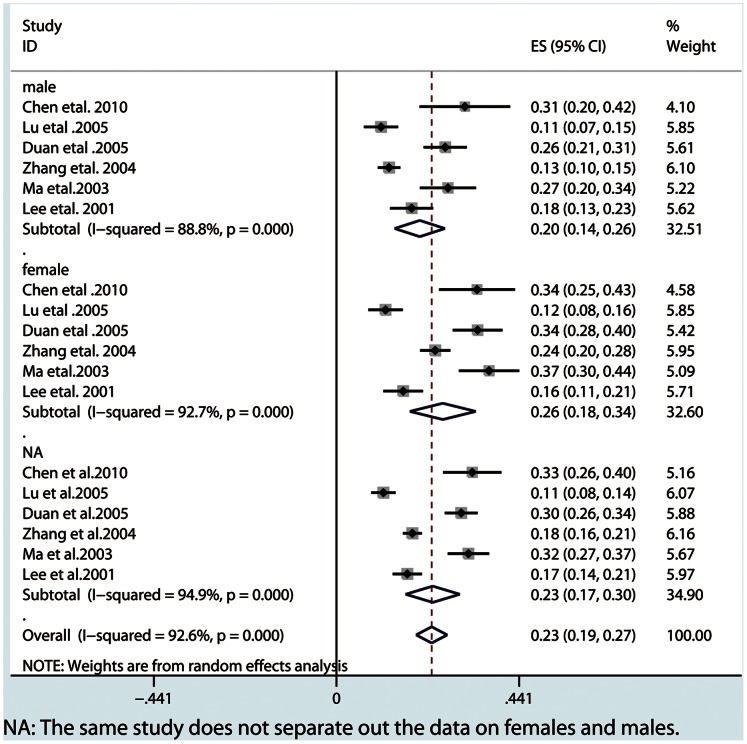
The overall 12-month prevalence in all the MDD patients and subgroups by gender.

**Figure 4:**
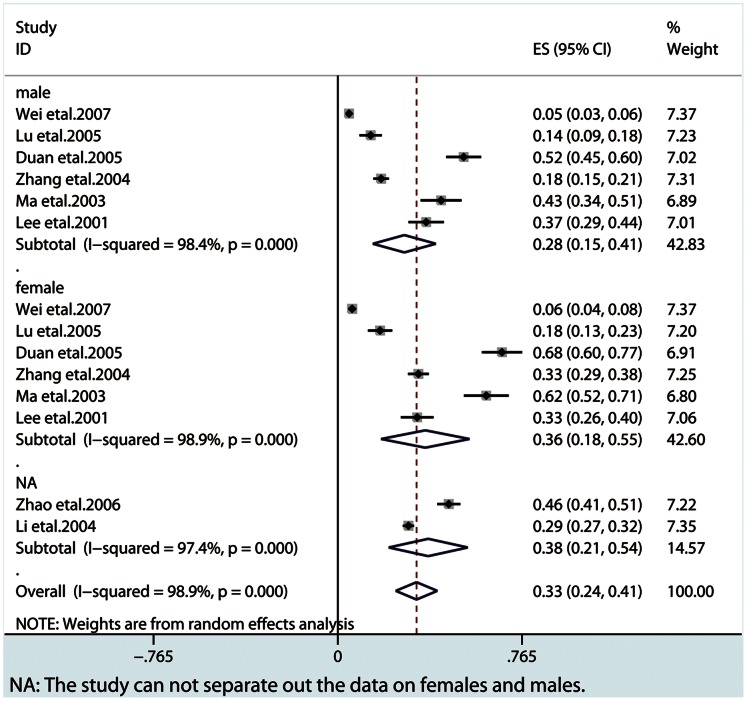
The overall lifetime prevalence in all the MDD patients and subgroups by gender.

**Figure 5:**
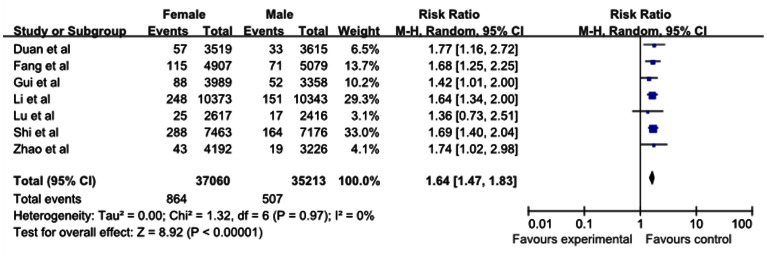
The risk of current prevalence of MDD among females compared with males in mainland China.

Our meta-analysis showed that the lifetime prevalence of MDD was 3.3% (95% CI: 2.4–4.1), the 12-month prevalence was 2.3% (95% CI: 1.8–3.4) and the current prevalence was 1.6% (95% CI: 1.2–1.9). From subgroup analysis by gender, females showed a higher prevalence in three MDD periods (Figs. 2, 3, 4 and Table 2). A higher current prevalence of MDD was found in females than males (OR = 1.64, 95%CI: 1.47–1.83) (Fig. 5).

**Table 2:** Overall and mean prevalence of MDD in subgroups.

Items	N	Case	Population	Prevalence (%)	95%CI
**Current**					
Overall	14	2,543	163,998	1.6	1.2–1.9
Male	7	507	35,213	1.3	0.8–1.7
Female	7	864	37,060	2.1	1.3–2.8
Urban	4	284	16,783	1.7	0.8–2.7
Rural	4	815	35,976	2.0	1.2–2.9
SCID	9	1,974	112,836	1.8	1.2–2.4
CIDI	5	569	51,162	1.1	0.4–1.8
**12-months**					
Overall	6	841	37,962	2.3	1.8–5.5
Male	6	340	19,631	2.6	1.8–3.4
Female	6	501	18,331	2.0	1.4–2.6
**Lifetime**					
Overall	8	2,334	81,846	3.3	2.4–4.1
Female	6	813	27,152	3.6	1.8–5.5
Male	6	571	26,560	2.8	1.5–4.1

We also stratified the included studies based on living area and the tools utilized for the current prevalence determination. The prevalence of MDD was significantly different in both areas and depending on the diagnostic tool (Table 2). It was found that the current prevalence of MDD provided from four areas was significantly higher in rural areas (2.0%, 95% CI: 1.2–2.9) than in urban areas (1.7%, 95% CI: 0.8–2.7). Subgroup analyses according to the use of standardized diagnostic tools revealed that the current prevalence of MDD was 1.1% (95% CI: 0.4–1.8) with the CIDI tool based on the data from five studies and 1.8% (95% CI: 1.2–2.4) with the SCID tool from nine studies (Table 2).

## Discussion

In this systematic review, 17 studies were included with a total number of 176,435 subjects. Compared with males, we found that females were at greater risk of MDD at the three different periods. Interestingly, our results showed that a relatively higher level of MDD risk was found among rural residents. In addition, a higher prevalence of MDD was found with the diagnoses using the SCID tool.

The national surveys in China have not been carried out since 1993. Thus, no reliable data on the prevalence of depression in mainland China are available. This review was the first meta-analysis for estimating the prevalence of MDD in mainland China. Our result showed that the current prevalence of MDD in mainland China is lower than in Asia including Japan (2.9%) [39] and in the United States (6.6%) [1]. Comparing the 12-month MDD prevalence rate of 2.3% in the multi-racial mainland Chinese population with the 17 countries that participated in the WHO World Mental Health (WMH) Survey, the result of the meta-analysis showed that the 12-month rate is lower than those in developing countries (4.0–10.4%) and are similar to that in Japan (2.2%) [40]. A similar result of lifetime prevalence rates was found in a large-scale epidemiological survey administering face-to-face CIDI interviews in 10 countries across North America, Latin America, Europe, and Asia (Japan), which included the results of lifetime prevalence estimates of major depressive episode (MDE) varying from 3% in Japan to 16.9% in the USA, with the majority of countries in the range of 8–12% [41]. Because of the differences in the methodology of the different studies, it is difficult to explain this regional variation [42]. It is possible that some degree of under-reporting occurred from those who might be unwilling to reveal emotional problems to an interviewer, as suggested by a number of researchers, and from the tendency of the Chinese towards somatization (where individuals persistently complain of physical rather than psychiatric symptoms) [43]–[45]. Furthermore, the cross-cultural reliability and validity of CIDI remain unclear because the validation exercises have been completed almost entirely in Western countries [46]–[48]. Apart from that, the true regional variability may be reflected by differences in the epidemiology of MDD.

Earlier epidemiological studies have shown that socio-demographic characteristics are important factors associated with the rates of mental disorders worldwide such as gender, age and race/ethnicity [49]. In particular, epidemiological surveys have consistently documented that the rates of anxiety and mood disorders were higher among women than men [50], [51]. Our results also indicated that the female gender was at a higher risk of MDD, which was consistent with the findings from the meta-analysis of the Iranian survey on gender difference in the current prevalence of MDD [52]. Unlike men, most of women have various reproductive or life stages, such as premenses, pregnancy, postpartum, and menopause. It has been suggested that biological and psychosocial factors lead to a higher vulnerability of women to major depression [53]. The hypothesis of social and cultural roles and/or some biological susceptibility in society would be consistent with our findings that women have higher rates of MDD.

In general, urban areas seem to be linked to a higher risk of mental health disorders, particularly depressive disorders [54]. On the contrary, we found that the current prevalence of MDD was higher in rural than urban areas, which is similar to a Belgian report [55]. It seems that the strength of the association with urbanization compared to other factors associated with the prevalence of psychiatric disorders, such as being unmarried or childhood abuse is limited [56]. Additionally, the inclusion of alternative measures of social stratification may be a reason that MDD is more prevalent in rural areas. The social and cultural limits induced by social stratification and the incapacity to use suitable skills to confront stress may underlie the higher prevalence of psychiatric disorders in rural areas [57]. On the other hand, the higher prevalence in rural areas might be explained by rural-to-urban migration in China. Thirteen percent of all rural families have at least of one family member who migrated to urban areas in the national scope [58]. A hypothesis might be that healthy people have migrated to the cities, while older and unhealthy people have stayed in the rural areas.

The present study did not show good concordance between diagnoses based on the CIDI and those based on the SCID results, which was different from the blinded clinical study with a probability subsample of World Mental Health (WMH) survey in four WMH countries [59]. However, the WHO WMH surveys have pointed out that the accuracy of CIDI diagnoses could be worse in non-Western countries, from a study in 17 countries. One distinct possibility is that there may be a weak relationship between CIDI symptoms and descriptions in non-Western cultures, or a greater reluctance to endorse emotional problems in countries with shorter traditions of free speech and anonymous public opinion surveying [47]. It is not clear that the deviations in the results of the meta-analysis are associated with the different methods or diagnostic tools, or sample population or the quality of the studies.

Several potential limitations of this study should be considered. First, we could only obtain data from 12 provinces and two municipalities in mainland China. The others without data include the provinces at the borders of China that suffer from lower socioeconomic status and more minorities, such as Xizang and Xinjiang. But the data include metropolitan cities, for example Guangzhou, Beijing. Thus, there needs to be epidemiological surveying of the prevalence of MDD from other provinces in mainland China. Second, the psychiatric epidemiological surveys of mainland China have adopted internationally recognized diagnostic criteria and standardized interview instruments at the beginning with 2000. But the 65 studies referred to the mental disorders studies, which did not provide individual MDD data, were found so that they could not be included in the meta-analysis. Additionally, the prevalence of MDD varied in different year period, age group, gender, diagnosis criteria and other possible factors (economic status, education level etc), based on the results of this manuscript and other papers. It is better to estimate the adjusted pool and subgroup prevalence of current MDD, after adjusting the above possible risk factors by multiple statistical models. But not all necessary information could be obtained from all included studies, adjusting the possible risk factors by multiple statistical models or adjusting the weight of these studies could not perform. A third limitation is that the results of the meta-analysis in some studies may be influenced by cross-cultural issues. DSM-IV diagnostic categories and criteria are widely used in clinical and research studies in China now, but the culture-specific ways of experiencing and manifesting psychological symptoms might make these categories and criteria less valid in China than other countries [60]. Because of the lack of clear biological markers for the mental disorders, the diagnoses are made on the basis of information provided by the target individuals and their associates. However, the individuals with debilitating conditions who due to education, language, culture, or other reasons, such as experience, express negative states in non-traditional ways might be excluded by the highly structured questions [60]. The diagnostic instruments, sampling methods and study design may introduce a potential bias to the observed heterogeneity in prevalence rates, genre, location, and tool differences in the meta-analysis. Finally, a further limitation is that we limited the studies published in English and Chinese, and the exclusion of unpublished studies which could have led to the omission of some articles about the prevalence of MDD published in different languages or not indexed by electronic databases mentioned in the introduction.

In conclusion, this study is the first meta-analysis conducted on the prevalence of MDD in mainland China. The results show that the prevalence of MDD in the general population of mainland China is lower than in other countries. A higher prevalence of MDD is found in females, rural areas, and with the SCID diagnostic tool. However, more epidemiological studies are required to further confirm the results.
